# Dietary protein restriction deciphers new relationships between lifespan, fecundity and activity levels in fruit flies *Drosophila melanogaster*

**DOI:** 10.1038/s41598-020-66372-4

**Published:** 2020-06-22

**Authors:** Sudhakar Krittika, Pankaj Yadav

**Affiliations:** Fly Laboratory # 210, Anusandhan Kendra-II, School of Chemical & Biotechnology, SASTRA Deemed to be University, Thanjavur, 613401 Tamil Nadu India

**Keywords:** Ageing, Metabolism

## Abstract

*Drosophila melanogaster* has been used in Diet Restriction (DR) studies for a few decades now, due to easy diet implementation and its short lifespan. Since the concentration of protein determines the trade-offs between lifespan and fecundity, it is important to understand the level of protein and the extent of its influence on lifespan, fecundity and activity of fruit flies. In this study, we intend to assess the effect of a series of protein restricted diets from age 1 day of the adult fly on these traits to understand the possible variations in trade-off across tested diets. We found that lifespan under different protein concentrations remains unaltered, even though protein restricted diets exerted an age-specific influence on fecundity. Interestingly, there was no difference in lifetime activity of the flies in most of the tested protein restricted (PR) diets, even though a sex-dependent influence of protein concentrations was observed. Additionally, we report that not all concentrations of PR diet increase activity, thereby suggesting that the correlation between lifespan and the lifetime activity can be challenged under protein-restricted condition. Therefore, the PR does not need to exert its effect on lifespan and fecundity only but can also influence activity levels of the flies, thereby emphasizing the role of nutrient allotment between lifespan, fecundity and activity.

## Introduction

Lifespan and fecundity are major fitness parameters that can assess the rate of aging and healthspan of organisms. It is suggested that lifespan and healthspan are no longer equivalent parameters and hence do not allow us to use lifespan alone to assess aging and fitness of an individual^1^. For the past two decades, studies on the role of nutrition in the regulation of fitness and its related traits have been studied to understand the intricate process of aging. Nutritional studies employ techniques like calorie restriction, Diet Restriction (DR; henceforth), food dilution, intermittent feeding, etc., for a long time in a variety of animal models such as mice, fruit flies and nematodes^2–4^. Since the validity of calorie restriction and food dilution has been debatable^5,6^, intermittent feeding and DR (involving restriction of one or more nutrients in the food, without causing malnutrition) have gained momentum in nutritional geometry studies. Numerous DR and aging studies have been done using fruit flies *Drosophila melanogaster* because of their ability to cater to a wide range of researches due to its shorter lifespan, well-understood genetics, and the presence of many equivalent genes in humans.

Implementing reduced yeast (protein source in fly food) can be termed as Protein Restriction (PR; a type of DR). Several studies have shown that PR can extend lifespan and also result in a possible trade-off in fecundity^2,5^. PR imposed on fruit flies *D. melanogaster* enables us to assess the effect of protein alone on fitness and fitness-related traits. Apart from the interplay between lifespan and fecundity upon DR, the locomotor activity of flies can also be used to assess the state of functional senescence (reviewed in^7^). Upon protein limitation, the flies can exhibit increased activity^4,8^ either as a result of foraging behavior towards higher nutrient availability^9,10^ or due to reduced toxicity because of restricted protein^11^. Although DR effect of enhancing the fly’s activity has been studied over a short time^8^, assessing the long-term effect of DR on the activity of flies and questioning whether the protein-restricted diet can improve the lifetime activity of the flies formed the basis of this investigation. Since various studies have reported evidence for^12–14^ and against^15–17^ the concept of inverse correlation between lifespan and metabolic rate (or physical activity), the physical activity of the flies and thereby, their metabolic rate can be influenced by various environmental conditions. This study aims to understand better the importance of PR diets on the adult flies in terms of (i) lifespan, fecundity and activity, and (ii) to define the reference levels of malnutrition and saturation point of protein concentration below which it could be detrimental for the flies’ survival.

The current study depicts an overlooked influence of a series of PR diets in enhancing the lifetime activity of flies, alongside influencing their lifespan and fecundity (here we measured fecundity as the number of eggs laid). We report that restricting protein for the flies does not need to always cause lifespan extension (LE; henceforth) and reduced fecundity, but can exhibit an unaltered lifespan if PR is implemented at age 1 day of the adult flies. In addition to this, most of the tested PR yielded a control-like effect on lifespan, fecundity and lifetime activity levels, showing that none of the tested concentrations were detrimental. Interestingly, our study shows that PR diets at this age can have a consistent positive effect on the three tested traits, showing that all these traits share the basis of nutrient allotment. Hence, the results of further studies might help to clarify the use of locomotor activity as a behavioral biomarker of aging.

## Results

### Effect of series of PR on lifespan

**Figure 1:**
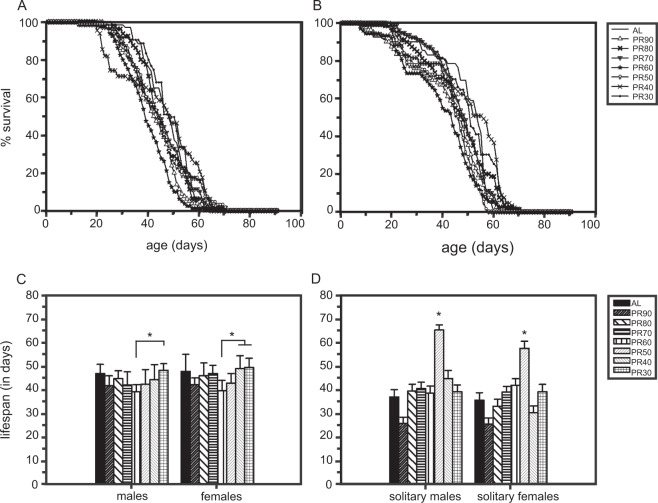
Effect of series of protein-restricted diet on the lifespan of fruit flies. Survivorship curves of males (**A**) and females (**B**) under a series of PR’s (30% to 90%) and their average lifespan (**C**; housed in groups of 10 flies) shows no difference in the PR flies as compared to the control. The lifespan of flies in solitude (**D**; from activity assay) showed that PR50% alone showed an increased lifespan against the control. The *x*-axis in the graph represents the age of flies (**A**,**B**) and sex (**C**,**D**), while the *y*-axis denoted the percentage survival of flies (**A**,**B**) and lifespan in days (**C**,**D**) respectively. The error bars are denoted with standard error around the mean (SEM) and the asterisks indicate statistical significance (*p* < 0.05). A total of 80 flies were used in control and each of the experimental PR diets.

Two-way ANOVA on lifespan data revealed statistically significant effect of Diet (D; F_*7,112*_ = 6.64, p < 0.0001) and Sex (S; F_*1,112*_ = 4.38, p < 0.0387), but not their interaction (D × S; F_*7, 112*_ = 0.58, p = 0.77; Table 2). Post hoc multiple comparisons by Tukey’s Honestly Significant Difference (HSD) test revealed no LE upon PR, hence the entire tested PR exhibited lifespan similar to AL (*ad libitum*; control). The survivorship curves of males and females under different PR (Fig. 1A,B) and mean lifespan of the flies under the same (Fig. 1C) show internal differences among different PR’s (Fig. 1A–C). The average lifespan of the solitary flies upon PR showed a significant effect of D (F_7,457_ = 21.88, p < 0.0001) and S (F_1,457_ = 5.76, p < 0.0168), but not of D × S (F_7,457_ = 1.76, p = 0.09). The average lifespan of males and females at PR50% is significantly higher than that observed at AL and the rest of the tested PR (Fig. 1D).

**Table 1:** Diet composition and the corresponding PR manipulations for 1 L (liter).

Components	PR30%	PR40%	PR50%	PR60%	PR70%	PR80%	PR90%	AL (unaltered protein)
Agar (g)	12	12	12	12	12	12	12	12
Corn (g)	100	100	100	100	100	100	100	100
Sugar (g)	40	40	40	40	40	40	40	40
Yeast (g)	12	16	20	24	28	32	36	40
Benzoic acid (g/ml)^!^	1/10	1/10	1/10	1/ 10	1/10	1/10	1/10	1/10
Propionic acid (ml)	10	10	10	10	10	10	10	10

^!^1 gram of Methyl-p-hydroxybenzoate crystals dissolved in 10 ml of Ethanol.

**Table 2:** ANOVA details of lifespan and fecundity assays done under LD12:12 h.

Assay	Effect	SS error	*d.f*.	MS effect	*d.f. error*	MS error	*F*	*p*<
Lifespan assay	Diet (D)	2631.9	7	156.0	112	23.5	6.64	0.0001
	Sex (S)	2631.9	1	102.8	112	23.5	4.38	0.0387
	D × S	2631.9	7	13.6	112	23.5	0.58	0.7733
Lifespan (solitary flies)	Diet (D)	120416	7	5765.5	457	263.5	21.881	0.0001
	Sex (S)	120416	1	1516.5	457	263.5	5.755	0.0168
	D × S	120416	7	463.7	457	263.5	1.760	0.0935
Fecundity Average eggs	Diet (D)	9412.7	7	2809.6	16	588.3	4.78	0.0046
	Age (A)	21255.8	3	22228.4	48	442.8	50.19	0.0001
	A × D	21255.8	21	2545.0	48	442.8	5.75	0.0001

### Influence of PR on fecundity is prominent in the middle age of the flies

**Figure 2:**
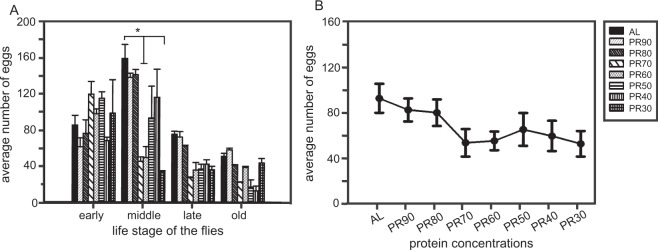
PR has an age-dependent effect on fecundity when imposed from age 1 day of the adult. Average egg output (**A**,**B**) during the assayed period of the fly’s lifespan showed that the average egg output is significantly lower in PR flies during the most reproductive phase of the flies. The *x*-axis denotes the age of flies (**A**) and protein concentrations (**B**), while the *y*-axis denotes the average egg output. All other details are the same as in Fig. 1. A total of 100 males and 100 females for control and each PR were employed in the experimental setup.

ANOVA on the average fecundity comparisons under AL and PR food within each age group showed a statistically significant effect of Diet (D; F_*7,16*_ = 4.78, p < 0.0046), Age (A; F_*3,48*_ = 50.19, p < 0.0001) and D × A (F_*21,48*_ = 5.75, p < 0.0001; Table 2; Fig. 2). Post hoc multiple comparisons using Tukey’s HSD test on the fecundity of PR flies during early, late and old age showed no significant difference as compared to that of AL. Interestingly at middle age, the egg output under PR30%, 60% and 70% is significantly lower than that of AL, while egg output of PR40%, 50%, 80% and 90% flies are similar to that of AL (Fig. 2A). The data also show that the average fecundity tends to decrease when the protein content decreases (Fig. 2B). Overall, the results on the average fecundity suggest that the effect of PR varies during the middle age (reproductive age) of the flies with respect to the AL, while it remains significantly unaltered at the early, late and old age.

### PR improves average lifetime activity of the flies

**Figure 3:**
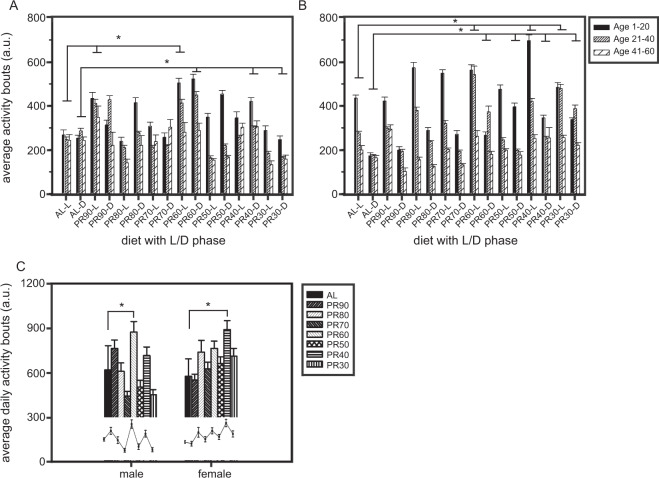
PR60% and PR40% increase the lifetime activity level of males and females respectively. Average activity level of control and PR males (**A**) and females (**B**) across a repeated measure of age shows increased activity at certain PR diets. The average lifetime activity of the flies (**C**) shows that PR60% (males) and PR40% (females) increase their activity levels against the AL food. The *x*-axis denotes diet with L/D (light/dark) phase (**A**,**B**) and sex (**C**), while the *y*-axis indicates the activity bouts in arbitrary units (a. u.). All other details are the same as in Fig. 1. A total of 32 flies for control and individual PR diets were assessed for their activity.

The activity levels of the male and female flies across age and sex were subjected to repeated measures ANOVA. ANOVA followed by post-hoc multiple comparisons by Tukey’s HSD test on the activity showed the significant effect of Diet (D; F_*7,606*_ = 54.55, p < 0.0001), Light/Dark phase (LD; F_*1,606*_ = 171.61, p < 0.0001), Sex (S; F_*1,606*_ = 14.24, p < 0.0001), Age (A; F_*2,1212*_ = 420.76, p < 0.0001) and all other interactions (Table 3). PR60% and PR90% (males) and PR30%, PR40% and PR60% (females) showed significantly higher light phase activity levels, while PR80% (males) showed lower activity as compared to AL (Fig. 3A,B). Surprisingly, during the dark phase also, the PR40% and PR60% (males) and PR30% to 60% (females) showed higher activity (Fig. 3A,B). Thus, PRs mediate increase in activity levels in both sexes, while it does not always result in higher activity post implementation.

**Table 3:** ANOVA details of locomotor activity assay performed under LD12:12 h.

Assay	Effect	SS error	*d.f*.	MS effect	*d.f. error*	MS error	*F*	*p*<
Average activity under LD phase	Diet (D)	6203540	7	558398	606	10237	54.55	0.0001
	Light-dark (LD)	6203540	1	1756741	606	10237	171.61	0.0001
	Sex (S)	6203540	1	145807	606	10237	14.24	0.0001
	D × LD	6203540	7	83559	606	10237	8.16	0.0001
	D × S	6203540	7	382341	606	10237	37.35	0.0001
	LD × S	6203540	1	2979091	606	10237	291.02	0.0001
	D × LD × S	6203540	7	55932	606	10237	5.46	0.0001
	Age (A)	12210787	2	4239088	1212	10075	420.76	0.0001
	A × D	12210787	14	170682	1212	10075	16.94	0.0001
	A × LD	12210787	2	319700	1212	10075	31.73	0.0001
	A × S	12210787	2	335281	1212	10075	33.28	0.0001
	A × D × LD	12210787	14	31839	1212	10075	3.16	0.0001
	A × D × S	12210787	14	82003	1212	10075	8014	0.0001
	A × LD × S	12210787	2	386182	1212	10075	38.33	0.0001
	A × D × LD × S	12210787	14	28190	1212	10075	2.8	0.0004
Average lifetime activity	Diet (D)	39637071	7	708570	496	79913	8.87	0.0001
	Sex (S)	39637071	1	589589	496	79913	7.38	0.0068
	D × S	39637071	7	447413	496	79913	5.59	0.0001

Further, to assess whether PR can mediate an overall increase in their lifetime activity apart from an age-dependent effect, we analyzed the average per day activity of the flies. Surprisingly, ANOVA on the average lifetime activity per day of flies imposed with AL and PR diets revealed the significant effect of D (F_*7,496*_ = 8.87, p < 0.0001), S (F_*1,496*_ = 7.38, p < 0.0068) and D × S (F_*7,496*_ = 5.59, p < 0.0001). Multiple comparisons by Tukey’s HSD test revealed that PR60% (males) and PR40% (females) alone exhibited an increased lifetime activity as compared to the AL (Fig. 3C). Thus, our results suggest that PR enhances the activity levels of male flies at PR60% and females at PR40%, but showed no difference in their lifespan in comparison to their AL counterparts (Fig. 1D). Thus, lifespan and activity levels of the flies can be influenced with mild changes in the diet protein levels, even though no negative outcomes have been observed.

## Discussion

Results of the current study show that protein restriction from age 1 day of the adult flies’ renders an unaltered lifespan, alongside age-dependent changes in fecundity and locomotor activity of fruit flies, *D. melanogaster*. Moreover, PR60% and PR40% enhance the lifetime activity levels of males and females respectively and most PR render fecundity similar to AL at early, middle, late and old ages.

Yeast in fly food can influence the lifespan and fecundity of the flies^2,5,18–20^. Increased protein consumption or a high protein diet may lead to a shortened lifespan and higher fecundity^21^. Since our experimental setup involved both the AL and experimental (PR) flies maintained on AL food in the pre-adult stage, we assume that all the flies would have consumed the same amount of food in their pre-adult stage. The unaltered lifespan after PR imposition from age 1 day of flies is contrary to many studies that reported LE upon DR^11,22,23^ and this can probably be explained because of the concentration of nutrients in our control diet itself. Interestingly, flies imposed with the PR from pre-adult stages showed LE [18, unpublished data], while no extension was observed when implemented from age 1 day, thereby showing that LE is feasible but is highly age and duration specific. The AL food used in our lab was formerly designed in such a way that it does not influence lifespan or fecundity and hence its constituents might be optimum by themselves, therefore PR in this respect is not yielding any LE.

The results of our study showed that the average fecundity of flies under PR30%, 60% and 70% were lower during the reproductive (middle) age of the flies (Fig. 2A). The same results were not seen within the early, late and old age groups: here the PR diets resulted in similar fecundity to that of AL. The reason for this not known, but suggests age dependent consequences of fecundity under PR. Having said this, one can observe that the lifespan of the virgin flies is similar in their corresponding PR’s (where there was altered fecundity) and AL flies. A study on yeast deprived larvae (3^rd^ instar) exhibited lower fecundity and unaltered longevity as they developed as adults^18^, and this result closely resembles the lifespan conclusion of our results stating that PR (from 3^rd^ instar larval stage or age 1 of adult age) need not necessarily always alter lifespan. But in case of fecundity, the same study cannot be compared mostly because of the pre-adult protein restriction which is thought to highly benefit fecundity. Hence, a breakdown in the correlation between the lifespan and fecundity is evident when a series of PR diets are employed, and thus suggesting possibilities of variations in trade-offs between these traits similar to Lee *et al*.^24^. Since male flies’ presence in the vial throughout the experimental period can enable the female flies to exhibit increased reproductive potential^25^ as compared to employing previously mated females alone, our experimental setup involved the same to assess the fecundity in a better possible way.

Activity levels of the flies are thought to influence lifespan, wherein the latter is reported to be inversely correlated ^26^, [references therein] or not^27,28^ with metabolic rate^29^. Since varied conclusions on this correlation have been reported in *Drosophila*, it is still debatable as discussed earlier. Similar to certain studies^4,8,17^, our results showed enhanced activity levels of male flies at PR60% and females at PR40% as compared to that of AL flies (Fig. 3), while these higher levels of activity did not have any influence on their lifespan (Fig. 1C). This is similar to the results reported by some studies^4,8,30^, but is also interesting that our study reports that not all PR concentration confer increased activity. Since the same PR diets mediate sex-based activity levels apart from age-specific effects, it adds evidence to conclude that activity levels are dependent on protein levels and sex^31^. The activity of AL and PR flies with respect to light and dark phase (Fig. 3A,B) was also measured to identify whether there is any significant difference in activity during the two phases. The flies show significant difference in their activity levels during the light and dark phase indicating that the morning is more responsive to environmental (light) changes than that seen in the afternoon or evening oscillator^32,33^. But since the majority of the morning peak and evening peak falls within the light phase, there is higher activity in this phase as compared to the other. However, it should be noted that the morning and evening oscillators can perform each other’s role in certain light conditions like natural environment, under dim or bright light^34,35^ etc.

Moreover, at old age, the flies show no difference in their activity levels between the LD phases and this can be thought to be the effect of age and probably an aged clock^36^. There might be a couple of possible reasons and since the pigment dispersing factor (PDF) is a key player in maintaining the rhythmicity and peaks of activity across age^37^. It might play a major role in regulating the activity bouts in the light/dark phases as well, similar to the expression levels of *clk* (clock), *per* (period), *tim* (timeless) upon DR^30^. Therefore, no significant difference in activity and lifespan of PR fed flies is observed, even though lifespan differences exist between flies housed in groups and in solitude. This could be because flight behavior in grouped flies housed in vials might be the cause of lower lifespan as compared to flies housed in solitary conditions^38,39^. It is also not surprising that restricting flight (due to the locomotor tube size) probably increased the lifespan of PR50% flies because of the reduced oxidative damage in the muscles that facilitate flight^38,39^. Hence, there might not be necessarily a trade-off between these two traits, like that reported between fecundity and lifespan.

Even though it is well studied that DR increases locomotor activity and lifespan, the correlation between these factors is debatable. The present study is contrary to the results of certain studies in fruit flies^15,40^ and house flies^13^ that state that there exists no correlation between these two traits either in a population or in solitary conditions as discussed earlier. This can be attributed to the traits themselves or due to the changes in fat metabolism^8^ and mitochondrial energy allocation^41,42^ upon DR. However, the presence or absence of a positive correlation has to be validated in the absence of stress conditions.

## Conclusion

Protein restriction studies have benefited in enhancing our understanding of metabolic, physiological and behavioral response of fruit flies to the changing nutritional environment. Our study reports that PR does not necessarily bring out LE, and hence the composition of control food (based on which the manipulations for studies are done) in different labs has to be validated. Moreover, since the effect of PR on the fecundity is dependent on the age and duration of PR imposition, certain PR-flies respond with lower fecundity during the most reproductive age of the flies (age 11–13), and exhibits control-like fecundity at other tested ages. Some levels of PR improve age-dependent activity levels and lifespan in solitary flies; thus, PR does not decrease lifespan. This study shows that imposing PR from the first day of adult life has no detrimental effect on lifespan and activity, even though a mild decrease in fecundity can be observed at three PR levels. Therefore, none of the PR diets can be considered as inducing malnutrition. Thus, most of the tested protein concentrations qualify as an effective PR diet and mediates an AL-like effect on lifespan (grouped), fecundity and lifetime activity of the flies.

## Methods

### Fly stock maintenance and fly culture

Wild-type strain of fruit flies, *D. melanogaster* (*Canton S*-CS) was obtained from late Prof. Vijay Kumar Sharma, Chronobiology Lab, JNCASR, Bangalore, India. The maintenance protocol of the fly stocks is similar to the one described elsewhere^43^. The flies were maintained on a banana–jaggery medium (fly food) inside a plexi glass cage of length-25 cm, breadth-20 cm and height-15 cm. During the experiment, the flies were maintained on their respective control and protein-restricted corn-sugar medium under a constant temperature of 25 °C (±0.5 °C), Light/Dark 12 h:12 h cycles, humidity (70 ± 5%). The agar from HIMEDIA and instant dry yeast from Gloripan were used for this study for fly food preparation media. The compositions of the control and the PR diets are given in Table 1.

### Lifespan assay

Egg collection for the assay was done in AL food and the freshly eclosed flies were later dispensed into a series of PR diets, such that the PR implementation is done at age 1 day of the adult fly. Freshly emerged fruit flies *D. melanogaster* from the vials were separated for males and females using mild CO_2_. The vials were maintained at ~25 °C temperature, ~70% humidity and LD12:12 h. A group of 10 flies (unmated males and females separately) was housed in each vial. Eight such vials were used for each of the PR diets and for the control setup [8 vials of 10 flies each × 2 sex × 8 setups (control + 7 PR)]. The control flies were fed with 100% protein or 40 gm yeast per liter of corn food, while the protein-restricted flies were fed with lower protein content (experimental-fed protein or yeast level ranging from 30% to 90% of that present in AL control; Table 1). Once the experiment was set up, the vials were assessed for the death of flies each day, and food change of the surviving flies was done on every 4^th^ day and this process was continued till the death of the last fly in each vial. The lifespan of flies was analyzed and calculated as the number of days they survived post–emergence: the data used in analyses were the average lifespan of the 10 flies in each vial (i.e. vial lifespan). In addition, we assayed the mean lifespan of the solitary flies (individual lifespan) that were housed as a part of the locomotor activity assay. The lifespan of a fly was calculated as the number of days it survived post-emergence.

### Fecundity assay

The experimental setup for fecundity assay (measured as the number of eggs laid) involved setting up of 10 male and 10 female flies per vial for the control and the respective PRs; wherein the same set of flies was used at 4 successive ages. The whole assay was carried out in LD (12 h light; 12 h dark) condition and a total of 10 such vials were set up for each PR and the control set, wherein the same set of flies were used for the assay. The total number of eggs laid by the flies in the vial during early age (1, 2 and 3 days), middle (reproductive) age (11, 12, 13 days), late age (21, 22, 23 days) and old age (31, 32, 33 days) were counted every 12 h during these ages (consecutive 3 days). At the time of egg counting, the flies were transferred into fresh food vials containing the respective AL and PR corn media and the old vials were used for egg counting. Apart from this, food was changed every fourth day between the consecutive egg counting days, to avoid unknown effects due to spoilage of food or its quality. The reproductive output at age 1, 2, 3 were considered as their average fecundity at age 2, and 11, 12, 13 as average output at age 12 and like-wise. The total egg output of the flies across the early to old age was calculated as the sum of the total number of the eggs laid during the age 1, 2, 3 (early age), age 11, 12, 13 (middle age) and so on.

### Activity assay

The lifetime activity assay of flies was performed using *Drosophila* activity monitors (DAM; Trikinetics, MA, USA). The egg collection for this assay was done like that of lifespan assay and the freshly eclosed flies were collected at 2 h interval and separated as males and females with mild CO_2_. Locomotor activity of the flies was monitored life-long under LD12:12 h wherein randomly 32 virgin males and females were loaded individually into glass tubes (5 mm diameter × 65 mm length) containing AL and the corresponding PR food (30% to 90% of the control yeast). Physical conditions such as temperature (~25 °C) and relative humidity (~70%) inside the recording cubicle were monitored every 5 minutes using *Drosophila* Environmental Monitor (DEnM) and were found to be stable. The time of the day on *x*-axis and the activity bouts on *y*-axis were plotted on the graphs. The lifespan of these solitary flies was also assayed as mentioned earlier, wherein the graph is plotted with males and females at the *x*-axis and lifespan (in days) at the *y*-axis.

### Statistical analysis

Lifespan data were subjected to ANOVA with the lifespan (in days) as dependent factor and Diet (D) and Sex (S) as independent factors. Fecundity assay involved consideration of D and Age (A) as the independent and repeated measure respectively, with the number of eggs laid as dependent factor. Similarly, activity data of fruit flies were also subjected to ANOVA with D, light-dark phase and S as independent factors and age as a repeated measure. The statistical significance is considered if *p* < 0.05 and the statistical analyses were performed using STATISTICA for Windows Release 7 (StatSoft Inc. 1995, 2004).
